# Morning Sickness

**Published:** 1860-08

**Authors:** Thomas Inman

**Affiliations:** Liverpool


### MORNING SICKNESS—ITS SIGNIFICANCE AS A SYMPTOM.

BY THOMAS INMAN, M. D., LIVERPOOL.

Few, if any, understand the significance of this symptom, though all are familiar with its occurrence. A host of questions may be asked concerning it. Why are pregnant women sick? Why in the morning? Why in the early months more than the later? Why does not this phenomena attend a distended bladder, or any other cause of enlargement of the abdomen, as frequently as it attends pregnancy? Does it occur in other complaints, and what have these in common with pregnancy? What is the proximate cause—is it to be sought in the stomach itself, the brain, the uterus, or all combined?

All pregnant women do not have it; some have it at one time, and not at another; it is rare among women in the country; and even among those who go from town, it is often found to cease, only to recur on returning to town. It is clear, then, that women are not sick because they are pregnant; something additional is required to produce the vomiting.

It is most common in the morning, yet it is not generally present so long as the recumbent position is maintained. As the brain is the organ most affected by change of posture, our attention is directed to that. Many cases are seen where vomiting occurs by assuming the erect position, and it is also a common sign of "water in the head." Few cases of this kind, however, have morning sickness; hence, a change in the cerebral circulation alone will not be sufficient to account for it.

We will next attempt to ascertain its causes by examining under what circumstances it occurs in males, children and elderly people. In several cases of men suffering under this symptom, it was the result of dyspepsia. In children, it is found as a tolerably sure indication of deficiency of digestive power in the stomach and body generally. When we inquire how much the condition of the uterus influences the vomiting, we find that it is not produced solely by enlargement of that organ, for it is not common when the womb is distended by an accumulation in consequence of an unruptured hymen; nor is it from pressure in the pelvis, either direct or indirect, for it is generally absent in ovarian dropsy: nor is it a common sign in cases of uterine tumors, etc.

From all these considerations, we may infer that "uterine sympathy" does not hold so prominent a place as the formation of a new being. Nor can we assign to either the most important rank, as neither produces this phenomenon, unless other conditions are present.

As the symptom does not occur in perfectly healthy and strong women, we infer that its occurrence depends upon some deterioration of vital power, and as this involves more or less deterioration in all the organs, we infer that in such cases there is a deficiency of vital power in the brain and stomach.

If this be true, the best remedies will be those which improve the general condition of the patient, which improve the steadiness of cerebral circulation, the tone of the stomach, and which deaden the sensibility of the organ which has been preternaturally increased by debility. If change of air cannot be adopted, then "diminish the daily work as far as possible, and increase the power to do it."

A case is related in illustration of this view, where a *man* had morning sickness for nine months every morning, about five minutes after rising. Regarding him as suffering from debility and overwork, he was ordered a glass of milk with some rum in it, to be taken before rising. Tincture of iron, and cod-liver oil were given through the day, with a nourishing diet and rest. The treatment was highly successful, and, after some four weeks, not having had any attacks of his old trouble, he one day worked too hard, and the next morning the sickness came on, and was again speedily relieved by the same treatment.

A similar plan must be adopted for pregnant women. They should take some food before rising, and allow sufficient time for this to have its influence. Perhaps milk combined with a stimulant is preferable; or hot coffee, cocoa, or tea. Where the sickness is very distressing, champagne or sparkling gooseberry wine answer well. Throughout the day, everything in the way of work must be carefully noted, in order that the patient may not overtask her powers. In bad cases, rest in bed may become necessary for a time.

Tonics, as steel, quinia, etc., are useful; opium, from its influence on the stomach and brain, is specially advantageous.

The preceding observations go far to explain those curious cases occasionally seen, where husbands suffer from this symptom as well as their wives; their sympathy being excited, soon produces faintness and sickness of the stomach. Such an explanation, however, will not apply to a case where a lady patient seriously asserted that in two out of four pregnancies, she was first made aware of her condition by the morning sickness of her husband.—*British Medical Journal*, *March* 24, 1860.

